# ID2 Regulates Macrophage Polarization and Mediates Methotrexate Responsiveness in Rheumatoid Arthritis

**DOI:** 10.1007/s10753-026-02527-1

**Published:** 2026-05-22

**Authors:** Lu Ye, Xiaomei Wang, Huaxiang Wu, Weihong Xu

**Affiliations:** 1 Department of Rheumatology, The Second Affiliated Hospital of Zhejiang University School of Medicine, 88 Jiefang Road, Hangzhou, 310009 China; 2 Department of Pathology, The Second Affiliated Hospital of Zhejiang University School of Medicine, 88 Jiefang Road, Hangzhou, 310009 China

**Keywords:** Rheumatoid arthritis, Methotrexate, Macrophage polarization, ID2, Single-cell RNA sequencing

## Abstract

Methotrexate (MTX) is the first-line treatment for rheumatoid arthritis (RA), but patient responses are highly variable. Macrophages are central drivers of RA inflammation, yet how monocyte/macrophage heterogeneity and transcriptional programs influence MTX responsiveness remains unclear. Single-cell RNA sequencing was performed on peripheral blood mononuclear cells from MTX-responsive and MTX-unresponsive RA patients to define immune composition and macrophage-like monocyte subsets. Polarization states were assessed using gene-signature scoring and pseudotime analysis. The role of inhibitor of differentiation 2 (ID2) was examined in LPS-stimulated RA patient–derived macrophages using gain- and loss-of-function approaches, cytokine assays and chromatin immunoprecipitation assays. Although overall immune cell distributions were similar between groups, macrophage-like monocytes from MTX-unresponsive patients exhibited higher M1 polarization scores. PLBD1⁺ and CDKN1A⁺ macrophage-like monocytes were enriched in MTX-unresponsive patients, whereas RLIM⁺ macrophage-like monocytes were reduced. Integrative analyses identified ID2 as a regulator associated with MTX resistance. ID2 expression was elevated in macrophage-like monocytes from MTX-unresponsive patients and positively correlated with M1 polarization. In vitro, MTX suppressed ID2 expression and inflammatory activation. ID2 knockdown reduced pro-inflammatory responses, whereas overexpression attenuated the inhibitory effects of MTX. ID2 was found to regulate EEF1A1 transcription. ID2 promotes pro-inflammatory macrophage polarization and may contribute to MTX resistance in RA.

**Supplementary Information:**

The online version contains supplementary material available at https://doi.org/10.1007/s10753-026-02527-1.

## Introduction

Rheumatoid arthritis (RA) is a persistent autoimmune disorder in which aberrant immune activity targets the synovial membrane, leading to joint pain, swelling, stiffness, warmth, and erythema [1, 2]. The disease most often presents as a symmetric polyarthritis involving the hands, wrists, feet, and knees [3]. Susceptibility to RA is influenced by multiple factors, including female sex, genetic predisposition, and tobacco use, which not only increases incidence but also aggravates disease severity. Additional contributors such as excess body weight, periodontal disease, environmental pollutants or silica exposure, and unhealthy dietary patterns further elevate risk, while alterations in the gut microbiota are emerging areas of investigation [4, 5].

Globally, RA affects approximately 0.5% of the population, with both point and period prevalence exceeding the overall average and showing notable regional variation [6]. Clinical management primarily aims to suppress inflammatory activity, alleviate pain, and preserve joint function through the use of methotrexate (MTX), biologics, or targeted small molecules, often supplemented by rehabilitation, behavioral modification, and surgical intervention when structural damage is advanced [7, 8]. However, diminished responsiveness to standard agents, including MTX resistance, increasingly limits therapeutic success [9]. Given that current approaches focus on disease control rather than eradication, there is a pressing need to identify innovative treatments grounded in a deeper mechanistic understanding of RA pathogenesis.

Macrophages are long-lived innate immune cells essential for debris clearance, pathogen elimination, antigen presentation, and orchestration of immune responses [10]. In healthy tissues, they contribute to repair and homeostasis, but dysregulation can promote chronic inflammatory conditions such as RA [11]. Within arthritic joints, macrophages amplify synovial inflammation and tissue destruction by secreting cytokines including tumor necrosis factor-α (TNF-α) and interleukin (IL)-1β, producing chemotactic factors, activating T lymphocytes, and releasing enzymes that degrade extracellular matrix. RA is characterized by a skewed balance toward pro-inflammatory M1 macrophages, accompanied by reduced anti-inflammatory M2 activity, resulting in sustained immune activation [12, 13]. Although macrophages exhibit considerable phenotypic flexibility, the mechanisms governing this imbalance, particularly in the context of drug resistance, remain insufficiently defined, making macrophage-directed modulation an attractive therapeutic avenue.

Accumulating evidence indicates that MTX modulates macrophage fate and functional states across diverse pathological settings. In the tumor microenvironment, MTX has been reported to redirect macrophages toward an antitumor phenotype through a glycogen synthase kinase-3β–dependent mechanism [14]. In the context of RA, MTX suppresses macrophage proinflammatory responses by inducing the expression of the anti-inflammatory regulator A20 [15]. In addition, MTX has been shown to preferentially eliminate human pro-inflammatory macrophages via a thymidylate synthase–p53–mediated pathway [16]. Nevertheless, how MTX shapes macrophage homeostasis in RA and the molecular processes governing this regulation remain incompletely understood.

Inhibitor of differentiation 2 (ID2) is a helix–loop–helix transcriptional regulator that functions as a dominant-negative partner to other family members by forming non-DNA-binding heterodimers. This protein is integral to the maturation and lineage commitment of diverse immune cell populations [17, 18]. Elevated ID2 expression has been linked to worsened RA outcomes through enhanced interferon-γ production by CD4⁺ T cells [19]. Notably, ID2 transcripts increase substantially during the differentiation of myeloid progenitors into granulocytes or macrophages in several leukemia-derived cell models, suggesting a role in shaping macrophage fate [20]. Despite these observations, it remains unresolved whether ID2 contributes to RA progression by directly influencing macrophage polarization and function.

In this study, peripheral blood mononuclear cells (PBMCs) were isolated from RA patients who were either responsive or unresponsive to MTX therapy and subjected to single-cell RNA sequencing (scRNA-seq). We focused on the monocyte/macrophage compartment, identifying distinct macrophage-like monocyte subsets and their polarization states in MTX-responsive versus MTX-unresponsive patients, and identifying ID2 as a candidate regulator associated with MTX responsiveness. To further investigate its functional role, macrophages were derived from RA PBMCs and stimulated with lipopolysaccharide (LPS) to induce M1 polarization, followed by MTX treatment. ID2 was subsequently silenced or overexpressed in LPS-activated macrophages to assess its contribution to MTX-mediated effects. Finally, we explored the molecular mechanisms by which ID2 regulates macrophage polarization in RA.

## Materials and Methods

### Human Samples

Peripheral blood samples were collected from patients diagnosed with RA. All participants fulfilled the 2010 American College of Rheumatology/European League Against Rheumatism diagnostic standards for RA. Disease activity was evaluated using the 28-joint Disease Activity Score (DAS28-CRP). For scRNA-seq analysis, blood samples were obtained from six patients, including three MTX-responsive and three MTX-unresponsive individuals, after at least three months of MTX therapy, during which nonsteroidal anti-inflammatory drugs (NSAIDs) were permitted and no other disease-modifying antirheumatic drugs (DMARDs), including biologic or targeted synthetic agents (such as JAK inhibitors), were used. MTX-unresponsive patients (UR_MTX, *N* = 3) had been treated with 15–20 mg/week of MTX for at least three months and exhibited a DAS28 decrease of less than 0.6, while MTX-responsive patients (R_MTX, *N* = 3) had received 15 mg/week of MTX for three months, demonstrated a DAS28 reduction of at least 1.2, and achieved a final DAS28 value below 3.2. For the monocyte-to-macrophage differentiation experiments, PBMC samples were collected from three independent RA patients who had not received any DMARDs, including conventional synthetic, biologic, or targeted synthetic agents (such as JAK inhibitors), and were treated with NSAIDs only. These samples were not derived from the scRNA-seq cohort. All enrolled patients exhibited moderate disease activity prior to treatment. Clinical characteristics of the patients are summarized in Supplementary Tables S1 and S2. This study was approved by the Ethics Committee of the Second Affiliated Hospital of Zhejiang University School of Medicine (approval number: 2025 − 1599). Written informed consent was obtained from all participants prior to enrollment, which detailed the study purpose, procedures, risks, confidentiality measures, and the right to withdraw at any time. All procedures involving human participants were conducted in accordance with the ethical standards of the institutional research committee and the Declaration of Helsinki.

## scRNA-seq and Data Preprocessing

PBMCs were separated from whole blood by density gradient centrifugation using Ficoll-Paque Plus (GE Healthcare) and subsequently washed with calcium- and magnesium-free phosphate-buffered saline (PBS). Erythrocytes were eliminated by incubating the cell suspension with 2 mL of GEXSCOPE^®^ red blood cell lysis buffer (Singleron) at 25 °C for 10 min. Samples were then centrifuged at 500 × g for 5 min, resuspended in PBS, and further processed by centrifugation at 400 × g for 5 min at 4 °C, after which the supernatant was discarded. Following red blood cell removal, PBMCs were collected by an additional centrifugation step at 400 × g for 10 min at 4 °C and resuspended in PBS to generate a single-cell suspension. Cell viability was assessed by Trypan Blue staining and microscopic examination.

Single-cell suspensions were adjusted to a concentration of 2 × 10⁵ cells/mL in PBS and loaded onto a microwell chip using the Singleron Matrix NEO^®^ Single Cell Processing System. Barcoding beads were retrieved from the chip, and captured mRNA was reverse transcribed to synthesize cDNA, followed by PCR amplification. The amplified products were fragmented and ligated with sequencing adapters to construct scRNA-seq libraries according to the GEXSCOPE^®^ Single Cell RNA Library Kit protocol (Singleron). Individual libraries were normalized to 4 nM, pooled, and sequenced on an Illumina NovaSeq 6000 platform with 150 bp paired-end reads.

Raw sequencing data were processed using the CeleScope pipeline (v1.9.0). Low-quality reads and adapter or poly-A sequences were removed with Cutadapt (v1.17), after which cell barcodes and unique molecular identifiers were extracted. Clean reads were aligned to the human reference genome (GRCh38, Ensembl v92) using STAR (v2.6.1a). Gene-level and UMI counts were generated with featureCounts (v2.0.1), producing expression matrices for downstream single-cell transcriptomic analyses.

## scRNA-seq Data Analysis

Single-cell gene expression matrices were processed using Seurat (v5.3.0). Initial quality control was performed to remove low-quality cells and potential technical artifacts. Cells with fewer than 200 detected genes or with more than 4,500 expressed genes (nFeature_RNA > 4,500) were excluded. In addition, cells exhibiting excessive mitochondrial gene expression (> 25% of total transcripts) were removed. Genes detected in fewer than five cells were also filtered out. The remaining high-quality cells were retained for downstream analyses. Raw read counts were log-normalized with the Log2Normalize method, after which the 2,000 most variable genes were identified using the variance-stabilizing transformation (VST) approach. The data were subsequently scaled and centered using the ‘ScaleData’ function. Principal component analysis (PCA) was then performed on the scaled matrix, and the top 20 principal components were selected for downstream analyses.

To minimize batch effects arising from sample heterogeneity or technical variation, cross-sample integration was conducted using canonical correlation analysis (CCA). Following integration, a shared nearest-neighbor graph was constructed using FindNeighbors (dims = 1:20), and unsupervised clustering was carried out with FindClusters at a resolution of 0.5 to identify distinct cellular subpopulations. Dimensionality reduction and visualization of global cellular architecture were performed using uniform manifold approximation and projection (UMAP) based on the same principal components.

Cell populations were manually annotated according to the expression patterns of well-established marker genes for each cell type, curated from the following publications [21–25]. Differential gene expression analysis between cell populations or experimental groups was performed using the ‘FindMarkers’ function with a minimum expression threshold of 25% of cells per group. Genes with an absolute log_2_FC > 0.25 and an adjusted *P*-value < 0.05 were considered differentially expressed.

Macrophage-like monocyte polarization states were further evaluated using a curated pro-inflammatory M1 macrophage gene signature (IL23, TNF, CXCL9, CXCL10, CXCL11, CD86, IL1A, IL1B, IL6, CCL5, IRF5, IRF1, CD40, IDO1, KYNU, CCR7) derived from a previous study [26]. The activity of this gene set in individual macrophages was quantified using AUCell (v1.28.0), enabling assessment of pro-inflammatory transcriptional programs at single-cell resolution.

To investigate cellular differentiation dynamics, pseudotime trajectory analysis was conducted using Monocle3 (v2.34.0). Cell state transitions were inferred and visualized in UMAP space. Genes associated with trajectory progression were identified using the ‘graph_test’ function, with significance defined by a q value < 0.05 and Moran’s I > 0.1.

## Macrophage Differentiation and Culture

PBMCs were suspended in PBS containing 1% human serum albumin, and 1 × 10⁷ cells were harvested by centrifugation at 1500 rpm for 5 min. The cell pellet was resuspended in 80 µL of MACS Running Buffer, incubated with 20 µL of NanoSep™ CD14 selection beads at 2–8 °C for 15 min, washed, and then resuspended in 1 mL of running buffer. The cell suspension was applied to a pre-equilibrated LS column placed in a MACS separator, washed twice with running buffer, and the flow-through was collected. The column was subsequently removed from the magnetic field, and the retained cells were eluted with 3 mL of running buffer using the supplied plunger. The eluted cells were centrifuged at 1500 rpm for 5 min, the supernatant was discarded, and the final pellet was resuspended in 1 mL of 1× DPBS for downstream assays.

To induce monocyte-to-macrophage differentiation, the enriched monocytes were resuspended in 50 mL of RPMI 1640 medium prewarmed to 37 °C and supplemented with 2 mM L-glutamine, 50 ng/mL M-CSF, 25 ng/mL interleukin-10, and 10% fetal bovine serum. The cell suspension was divided equally, with 25 mL seeded into each of two 75 cm² tissue culture flasks and incubated at 37 °C in a humidified atmosphere containing 5% CO₂ for 48 h. Cultures were then gently washed three times with 10 mL of warm RPMI 1640 to remove non-adherent cells, after which fresh complete medium was added and replaced every two days until the cells differentiated, expanded, and reached confluence, typically within one week.

## Plasmid Construction and Virus Production

The coding sequence of ID2 was cloned into the pLV3-CMV-3×FLAG-CopGFP-Puro vector, resulting in the plasmid named ID2 OE. Short hairpin RNAs (shRNAs) targeting ID2 were inserted into the pLKO.1 lentiviral backbone, generating plasmids referred to as shID2-1, shID2-2, and shID2-3. The primers used for plasmid construction are listed in Table 1. Lentivirus production was performed by Anti-hela Biotechnology (China), and the virus titer was measured using a Lentivirus Titer p24 ELISA Kit (L00938, GenScript, USA).

**Table 1:** Primers for plasmid construction.

Primer name	Sequence (5′-3′)
ID2-OE-F	TACGAACTCGGAATTCGCCACCATGAAAGCCTTCAGTCCCGTG
ID2-OE-R	CTTTGTAGTCGGATCCGCCACACAGTGCTTTGCTG
shID2-1-F	CCGGGACCACCCTCAACACGGATATCTCGAGATATCCGTGTTGAGGGTGGTCTTTTT
shID2-1-R	AATTAAAAAGACCACCCTCAACACGGATATCTCGAGATATCCGTGTTGAGGGTGGTC
shID2-2-F	CCGGCCCTTCTGAGTTAATGTCAAACTCGAGTTTGACATTAACTCAGAAGGGTTTTT
shID2-2-R	AATTAAAAACCCTTCTGAGTTAATGTCAAACTCGAGTTTGACATTAACTCAGAAGGG
shID2-3-F	CCGGAGCACTGTGTGGCTGAATAAGCTCGAGCTTATTCAGCCACACAGTGCTTTTTT
shID2-3-R	AATTAAAAAAGCACTGTGTGGCTGAATAAGCTCGAGCTTATTCAGCCACACAGTGCT
shNC-F	CCGGTTCTCCGAACGTGTCACGTTTCTCGAGAAACGTGACACGTTCGGAGAATTTTT
shNC-R	AATTAAAAATTCTCCGAACGTGTCACGTTTCTCGAGAAACGTGACACGTTCGGAGAA

### CCK-8 Assay

For CCK-8 assays, 3 × 10⁴ cells suspended in 0.2 mL full medium were inoculated into each well of 96-well plates. Following 24 h culture, each well was supplemented with 10 µL CCK-8 reagent and maintained for 1 h. Absorbance at 450 nm was recorded by a Multiskan SkyHigh spectrophotometer (A51119500C, Thermo Fisher Scientific).

## Western Blot

Cell or tissue samples were processed for total protein extraction using a protein lysis buffer (21-040-CV, Corning, USA) containing a protease inhibitor (329-98-6, Sigma-Aldrich, USA). The concentration of total protein was determined using a BCA Protein Assay Kit (23225, Thermo Fisher Scientific). Next, 20 µg of protein was separated by 10% SDS-PAGE and transferred to PVDF membranes. To block non-specific binding, the membranes were incubated with 5% non-fat milk in TBST. The membranes were then incubated overnight at 4 °C with primary antibodies. The following day, the membranes were washed three times with TBST and incubated with secondary antibodies at room temperature for 1 h. After three additional washes with TBST, the membranes were incubated with ECL substrate (NCM Biotech, P10100) for 2 min. The membranes were then exposed to X-ray film in a darkroom, followed by standard development and fixation. The films were scanned, and band intensities were quantified using ImageJ software (NIH, USA). The antibody information is listed in Table 2.

**Table 2:** Western blot antibody information.

Target	Species	Manufacturer	Catalog#	Dilution
CD80	Mouse	Thermo Fisher Scientific	TA812361	1:500
iNOS	Rabbit	Abcam, UK	ab3523	1:500
CD86	Mouse	Abcam	ab220188	1:500
ID2	Rabbit	Cell Signaling Technology, USA	#3431	1:500
Arg-1	Rabbit	Proteintech, China	16001-1-AP	1:500
CD206	Mouse	Proteintech	60143-1-Ig	1:500
EEF1A1	Rabbit	Proteintech	11402-1-AP	1:2000
Beta-Actin (Actin)	Rabbit	Proteintech	66009-1-Ig	1:500
HRP-Anti-Mouse IgG	Goat	Proteintech	SA00001-1	1:5000
HRP-Anti-Rabbit IgG	Goat	Proteintech	SA00001-2	1:5000

## Enzyme-linked Immunosorbent Assay (ELISA)

Cytokine levels in the conditioned media were measured using ELISA kits, according to the manufacturer’s instructions provided with each kit. The following kits were used: TNF-α (EK182, MULTI SCIENCES, China), IL-12p70 (EK112, MULTI SCIENCES), IL-10 (EK110, MULTI SCIENCES), and IFN-γ (CB14461-Hu, COIBO BIO, China). The microplate reader is the Multiskan SkyHigh spectrophotometer (A51119500C, Thermo Fisher Scientific).

### Quantitative PCR (qPCR)

Total RNA was extracted using the RaPure Total RNA Kit (Magen, China) and reverse transcribed into cDNA with the All-in-One First-Strand SuperMix (with dsDNase) (NY-Bio, China). qPCR was conducted using ChamQ SYBR Color qPCR Master Mix (Vazyme, China) on a QuantStudio3 machine (A28573, Thermo Fisher Scientific). The expression levels of target genes were normalized to 18 S rRNA and quantified using the 2^−ΔΔCt^ method. The primers used are listed in Table 3.

**Table 3:** Primers for qPCR.

Target gene	Forward primer (5′-3′)	Reverse primer (5′-3′)
ID2	GAAAAACAGCCTGTCGGACC	TGAGCTTGGAGTAGCAGTCG
iNOS	AGGGACAAGCCTACCCCTC	CTCATCTCCCGTCAGTTGGT
EEF1A1	GTCTGTCAAGGATGTTCGTCG	GGCAATCCAATACAGGGGC
Arg-1	TGGACAGACTAGGAATTGGCA	CCAGTCCGTCAACATCAAAACT
18 S rRNA	CGACGACCCATTCGAACGTCT	CTCTCCGGAATCGAA CCCTGA

### Chromatin Immunoprecipitation (ChIP) and ChIP-seq

Macrophages transfected with empty vector or ID2 OE plasmids were subjected to ChIP using a monoclonal anti-FLAG antibody (Sigma-Aldrich, USA, F1804) and the EpiTect ChIP OneDay Kit (Qiagen, Germany, #334471) according to the manufacturer’s instructions. Enriched DNA was quantified by RT-PCR or qPCR using primers listed in Table 4, with all experiments performed in triplicate.

For ChIP-seq, macrophages were collected 24 h after transfection, washed with PBS, and crosslinked with freshly prepared 1% formaldehyde for 7 min at room temperature. Crosslinking was quenched with 0.125 M glycine. Nuclei were isolated by sequential lysis and resuspended in shearing buffer containing protease inhibitors. Chromatin was fragmented using a Covaris E220 ultrasonicator (duty factor 5%, peak incident power 140 W, 200 cycles per burst at 4 °C), generating fragments of approximately 250–800 bp, as confirmed by agarose gel electrophoresis. 1% of the sheared chromatin was reserved as input.

For each replicate, 50 µg of chromatin was incubated overnight at 4 °C with 5 µg anti-FLAG antibody in immunoprecipitation buffer containing BSA-blocked Protein G Dynabeads (Thermo Fisher Scientific). Beads were washed extensively, and bound DNA was eluted using 1% SDS and 0.1 M NaHCO₃. Eluates were treated with RNase A and Proteinase K, followed by overnight reverse crosslinking at 65 °C. DNA was purified by phenol–chloroform extraction and isopropanol precipitation. ChIP-seq libraries were constructed using the NEBNext Ultra II DNA Library Prep Kit (New England Biolabs, USA, E7103). Library quality was assessed with a Fragment Analyzer™, and sequencing was performed on an Illumina HiSeq V4 platform using single-end 50 bp reads.

For data analysis, raw sequencing data in FASTQ format were processed using the Vazyme cloud analysis platform, including quality control, adapter trimming, alignment to the human reference genome (hg38), peak calling, and differential binding analysis. BigWig files were visualized with Integrative Genomics Viewer (IGV, v2.16.1). Differentially enriched peaks were identified based on thresholds of log_2_FC > 0.58 and adjusted *P*-value < 0.05.

**Table 4:** Primers for ChIP-qPCR.

Target gene	Forward primer (5′-3′)	Reverse primer (5′-3′)
EEF1A1	CAGTACACGACATCACTTTC	AGAACCCAGAGATCGCTGC

### Luciferase Assay

The promoter region of EEF1A1 (NM_001402.6) was inserted into the pGL4.20 luciferase reporter vector to generate the pGL4.20-EEF1A1 construct. To assess ID2 binding activity, HEK293T cells were co-transfected with either empty pGL4.20 or pGL4.20-EEF1A1 together with a control plasmid or an ID2 overexpression vector using Exfect 2000 Transfection Reagent (Vazyme, T202-01). After 24 hours, cells were collected, and luciferase activity was measured with the Dual-Luciferase Reporter Assay Kit (Vazyme, DL101-01) on a GloMax 20/20 luminometer (Promega, USA). Relative luciferase activity was expressed as the ratio of Firefly to Renilla luciferase signals. The following primers were used for pGL4.20-EEF1A1 construction: pGL4.20-EEF1A1-F: 5’-CTAACTGGCCGGTACCCTGTGTTCTGGCGGCAAACC-3’, pGL4.20-EEF1A1-R: 5’- TCTTGATATCCTCGAGTTGGGGAATCCCAGGGACCG-3’.

### Statistical Analysis

Unpaired two-tailed Student’s t-test was used to compare two groups, while one-way ANOVA followed by Tukey’s HSD test was used for comparisons among multiple groups. Data are presented as mean ± standard deviation (SD). The Mann–Whitney U test was used to compare baseline characteristics and disease activity between MTX responders and non-responders. A P value < 0.05 was considered statistically significant. Statistical analyses were performed using GraphPad Prism (v8.0).

## Results

### Single-cell Landscape of Macrophage-like Monocyte Subpopulations in MTX-responsive and MTX-unresponsive RA Patients

**Fig. 1:**
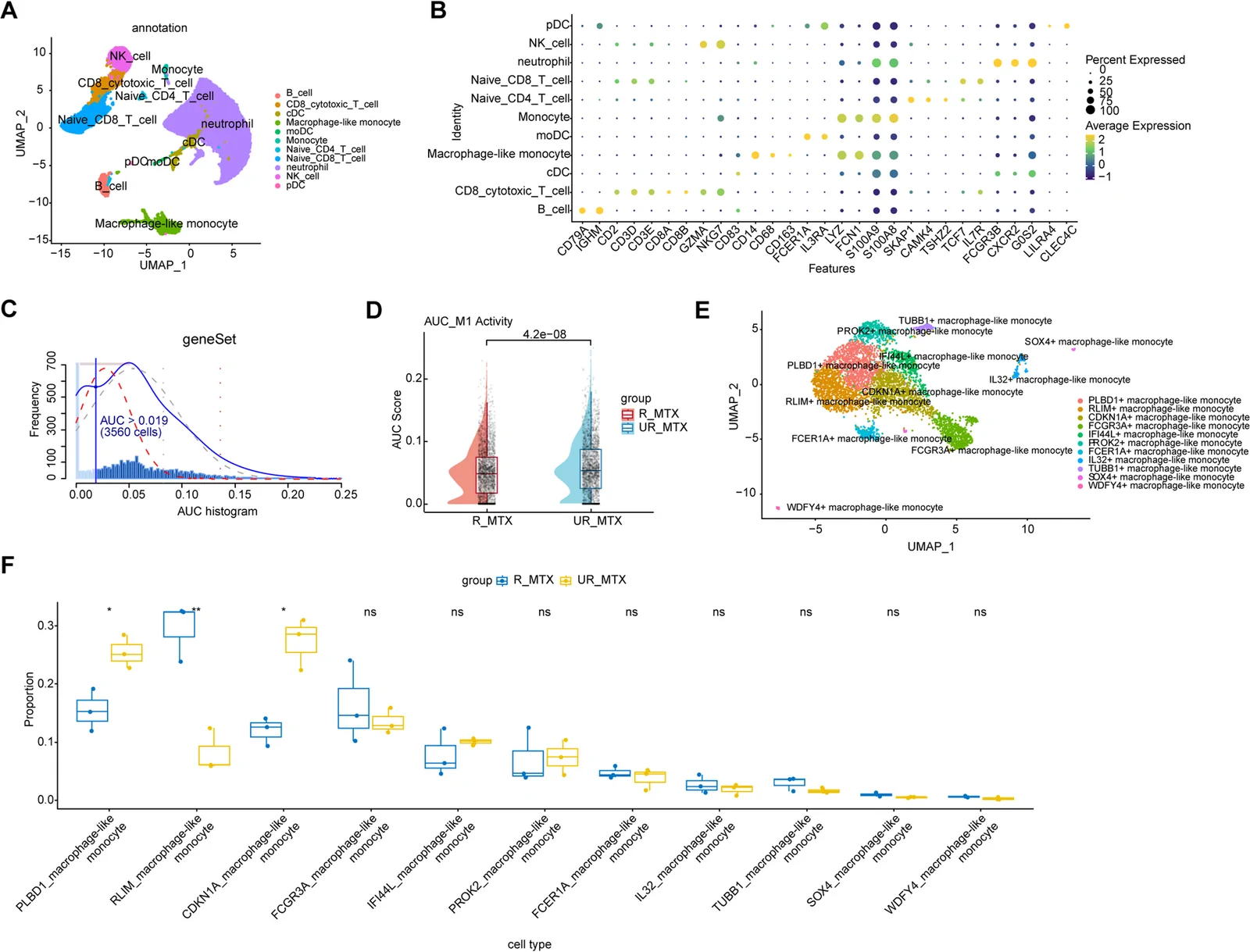
Single-cell landscape of PBMCs from MTX-responsive and MTX-unresponsive RA patients. (**A**) UMAP plot of PBMCs from MTX-responsive (R_MTX) and MTX-unresponsive (UR_MTX) patients showing 11 major immune cell populations annotated by canonical marker genes. (**B**) Dotplot showing selected marker gene expression across identified cell types. (**C**) AUCell scoring of inflammatory M1 macrophage gene signature across macrophage-like monocytes from R_MTX and UR_MTX patients. (**D**) Quantification of M1 scores in macrophage-like monocytes between groups. (**E**) UMAP plot of macrophage-like monocytes showing distinct subpopulations identified by signature genes. (**F**) Relative proportions of macrophage-like monocytes subpopulations in R_MTX versus UR_MTX patients. ^*^, *P* < 0.05; ^**^, *P* < 0.01; *ns*, not significant

We firstly compare the baseline characteristics and disease activity of RA between MTX responders and non-responders. The results demonstrated that the two groups were comparable in terms of age, disease duration, and pre-treatment DAS28-CRP. To characterize the molecular features in RA patients with different responses to MTX, scRNA-seq was performed on PBMCs isolated from MTX-responsive and MTX-unresponsive patients. Based on canonical PBMC marker genes, 11 major cell populations were identified, including B cells (CD79A, IGHM), CD8 cytotoxic T cells (CD8A, CD8B), conventional dendritic cells (cDCs; CD83), monocyte-derived dendritic cells (moDCs; FCER1A, IL3RA), monocytes (S100A8, S100A9, LYZ, FCN1), naïve CD4 T cells (SKAP1, CAMK4, TSHZ2), naïve CD8 T cells (CD2, CD3D, CD3E, IL7R, TCF7), neutrophils (FCGR3B, CXCR2, G0S2), NK cells (GZMA, NKG7), plasmacytoid dendritic cells (pDCs; IL3RA, CLEC4C) and a cluster expressing macrophage-associated markers (macrophage-like monocyte, CD14, CD68, CD163) (Fig. 1A, B). Considering that the samples used for scRNA-seq were derived from PBMCs, we referred to the cluster expressing macrophage-associated markers as macrophage-like monocytes. The overall distribution of these immune populations was comparable between the R_MTX and UR_MTX groups (Figure S1).

We next focused on the macrophage-like monocyte compartment and assessed their polarization status using an inflammatory M1 gene signature scored by the AUCell algorithm. Macrophage-like monocyte from MTX-unresponsive patients exhibited significantly higher M1 scores compared with those from MTX-responsive patients (Fig. 1C, D), suggesting that enhanced M1 polarization is associated with MTX resistance. Further subclustering identified 11 macrophage-like monocytes subsets characterized by the expression of PLBD1, RLIM, CDKN1A, FCGR3A, IFI44L, PROK2, FCER1A, IL32, TUBB1, SOX4, and WDFY4 (Fig. 1E and Figure S2 A). Among these subsets, PLBD1⁺ and CDKN1A⁺ clusters were less abundant in MTX-responsive patients, whereas RLIM⁺ clusters were reduced in MTX-unresponsive patients (Fig. 1F). Pseudotime analysis revealed that FCGR3A⁺ macrophage-like monocyte was the least differentiated, while PROK2⁺ and RLIM⁺ macrophage-like monocyte represented more terminally differentiated states (Figure S2 B, C). Together, these findings suggest that altered macrophage-like monocyte polarization and subpopulation distributions are associated with MTX responsiveness in RA.

### Identification of key regulators associated with MTX resistance in macrophage-like monocyte

**Fig. 2:**
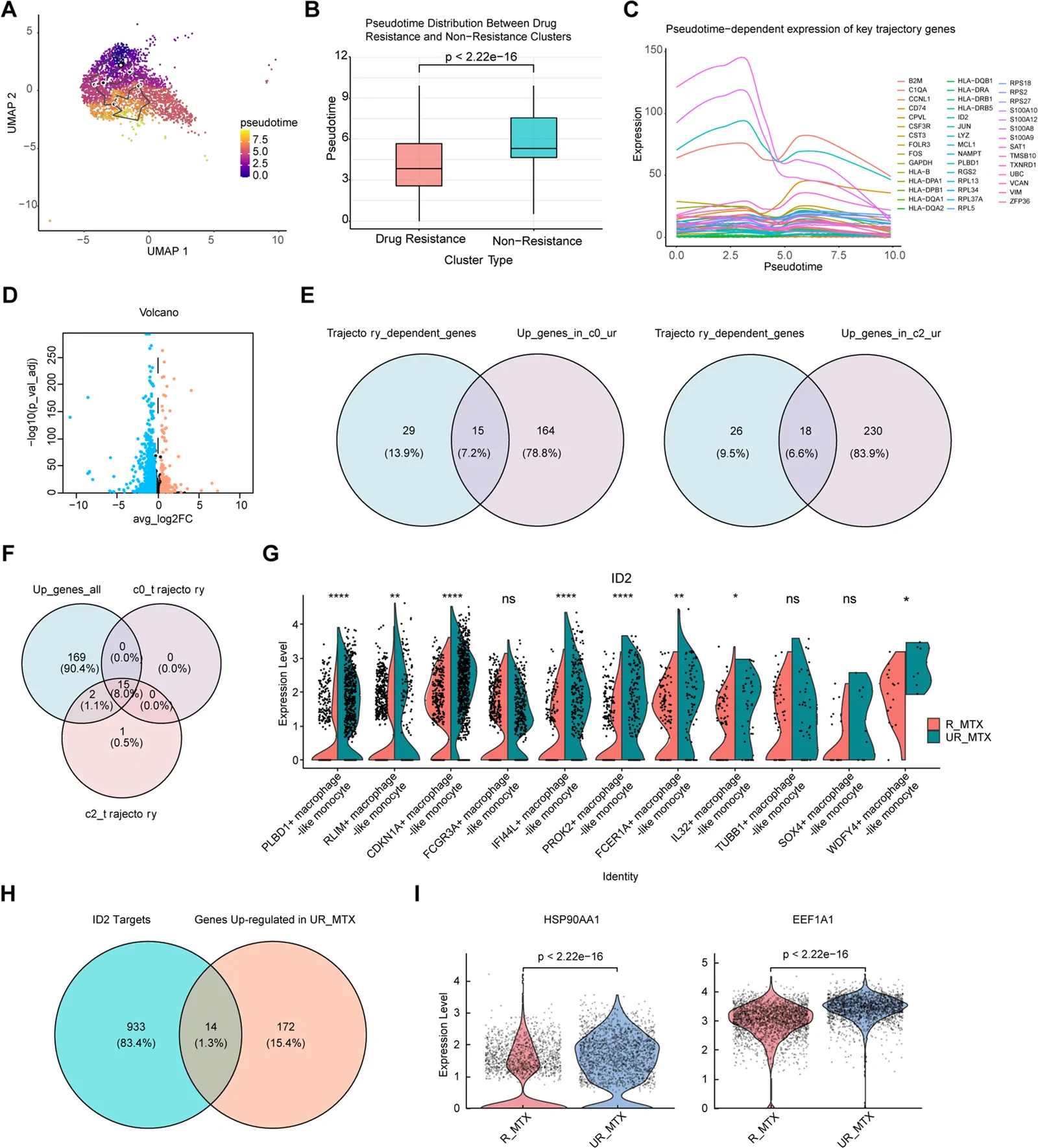
Identification of ID2 as a key regulator associated with MTX resistance in RA macrophage-like monocytes. (**A–C**) Pseudotime trajectories and related genes of PLBD1⁺, RLIM⁺, and CDKN1A⁺ macrophage-like monocytes in MTX-responsive and MTX-unresponsive patients. (**D**) Volcano plot indicating differentially expressed genes in all MTX-unresponsive macrophage-like monocytes. Red and blue colors represent upregulated and downregulated genes, respectively. (**E**,** F**) Venn diagram showing intersected genes upregulated in MTX-unresponsive macrophage-like monocytes subsets and trajectory-associated genes. (**G**) Violin plots showing ID2 expression across macrophage-like monocytes subpopulations in R_MTX and UR_MTX patients. (**H**) Intersection of putative ID2 target genes from TFLink with upregulated genes in MTX-unresponsive macrophage-like monocytes. (**I**) Violin plot indicating the expression of representative ID2 target genes HSP90AA1 and EEF1A1 in macrophage-like monocytes from R_MTX and UR_MTX patients. ^*^, *P* < 0.05; ^**^, *P* < 0.01; ^****^, *P* < 0.0001; *ns*, not significant

To explore the molecular mechanisms underlying differential MTX responses in macrophage-like monocyte, we further analyzed the three macrophage-like monocytes subpopulations that differed significantly between groups (PLBD1⁺, RLIM⁺, and CDKN1A⁺). Pseudotime trajectory analysis revealed marked differences in gene expression dynamics across these subsets between MTX-responsive and MTX-unresponsive patients (Fig. 2A–C).

To identify candidate regulatory genes, we screened genes upregulated in MTX-unresponsive PLBD1⁺ macrophage-like monocyte (C0, 179 genes), CDKN1A⁺ macrophage-like monocyte (C2, 248 genes), and all macrophage-like monocyte populations (186 genes) and intersected these with differentially expressed trajectory-associated genes (44 genes). This approach yielded 15 overlapping genes, including RPL37A, HLAB, UBC, S100A9, CCNL1, VIM, JUN, MCL1, NAMPT, RPL13, ID2, SAT1, S100A8, GAPDH, and S100A10 (Fig. 2D–F). Among these candidates, ID2 was selected for further investigation due to its known relevance to RA pathogenesis and monocyte/macrophage biology. Notably, ID2 expression was reduced in most macrophage-like monocyte subpopulations from MTX-responsive patients, with the exception of FCGR3A⁺, TUBB1⁺, and SOX4⁺ subsets (Fig. 2G).

To further explore ID2-mediated regulatory networks, putative ID2 target genes from the TFLink database were intersected with genes upregulated in MTX-unresponsive macrophage-like monocyte, identifying 14 candidate downstream targets, including CHD1, RPS1, ATF4, CD44, MECOM, UPP1, RPLP2, HSP90AA1, RPSA, RPS12, ETF1, EEF1A1, RPL24, and NXF1 (Fig. 2H). Among these, HSP90AA1 and EEF1A1 are known regulators of macrophage function and polarization [27, 28], and their expression was reduced in MTX-responsive macrophages in parallel with decreased ID2 expression (Fig. 2I). These findings suggest that ID2 may serve as a key regulator linking macrophage polarization to MTX responsiveness in RA.

### MTX suppresses ID2 expression and inhibits M1 polarization in LPS-stimulated RA macrophages

To determine whether MTX directly regulates ID2 expression in RA macrophages (RA-Mφ), monocytes isolated from RA PBMCs were differentiated into macrophages and stimulated with lipopolysaccharide (LPS; 1 µg/mL) to induce M1 polarization. ELISA confirmed that LPS significantly increased TNF-α secretion, validating effective pro-inflammatory activation (Figure S3 A). RA-Mφs were then treated with increasing concentrations of MTX. CCK-8 assays demonstrated that MTX concentrations ≥ 25 nM significantly reduced cell viability, with an estimated IC₅₀ of approximately 100 nM (Figure S3B); therefore, 100 nM MTX was used for subsequent experiments. ELISA further showed that this concentration markedly decreased TNF-α production in LPS-stimulated RA-Mφs (Figure S3 C), consistent with its anti-inflammatory effects.

**Fig. 3:**
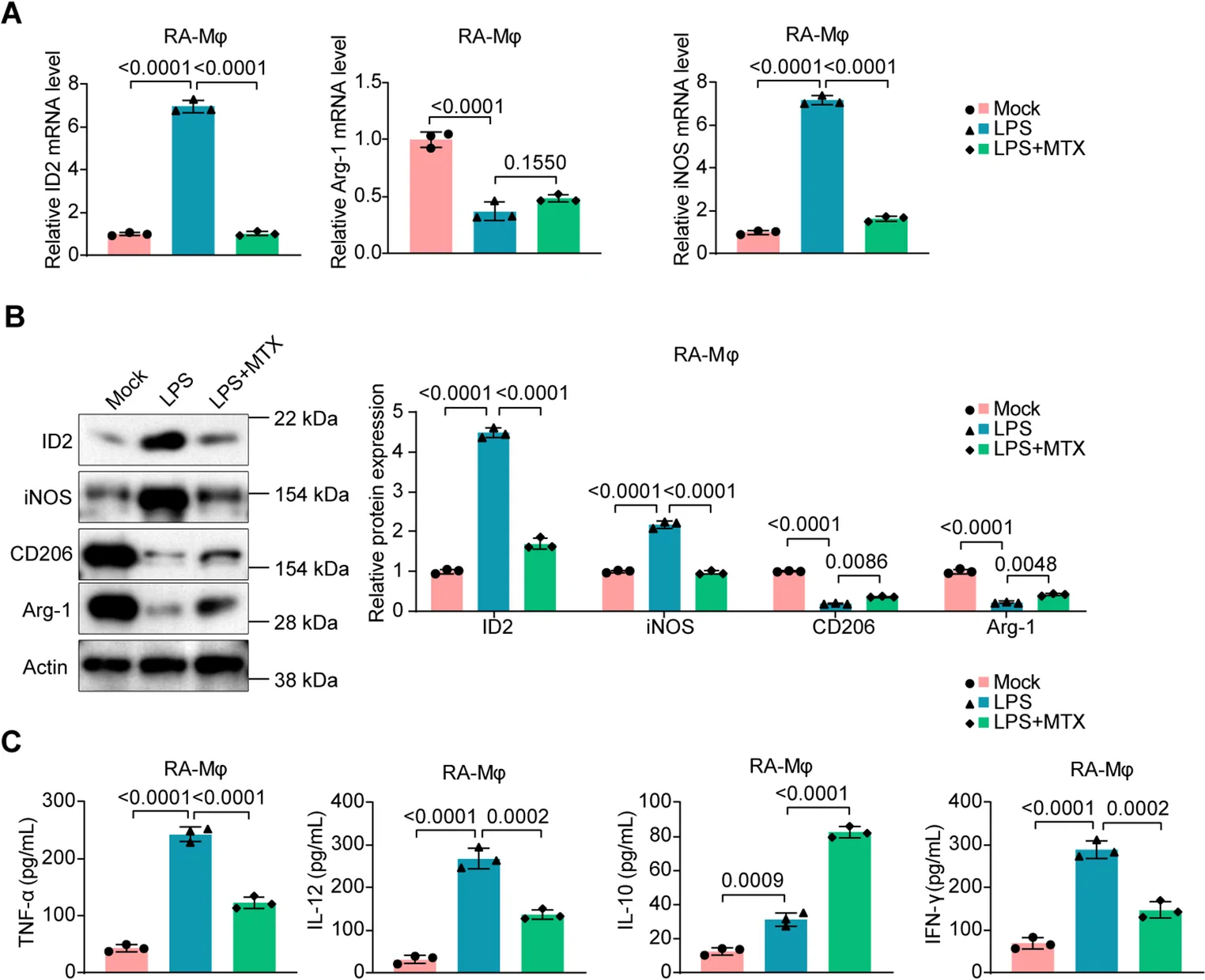
MTX suppresses ID2 expression and M1 polarization in LPS-stimulated RA macrophages. (**A**) qPCR analysis of ID2, iNOS, and Arg-1 in LPS-stimulated RA macrophages with or without MTX treatment. Mock defines PBS treatment. (**B**) Western blot showing ID2, iNOS, CD206, and Arg-1 expression under the same conditions. (**C**) ELISA quantification of pro-inflammatory (TNF-α, IL-12, IFN-γ) and anti-inflammatory (IL-10) cytokines

We next examined ID2 expression in LPS-stimulated RA-Mφs with or without MTX treatment. Both qPCR and Western blot analyses revealed that LPS markedly increased ID2 expression, whereas MTX treatment significantly suppressed ID2 levels (Fig. 3A, B). LPS-treated RA-Mφs exhibited increased secretion of pro-inflammatory cytokines (TNF-α, IL-12, IFN-γ), elevated expression of the M1 marker iNOS, and reduced levels of the anti-inflammatory cytokine IL-10 and M2 markers Arg-1 and CD206 (Fig. 3A–C). These effects were substantially reversed by MTX treatment. Collectively, these results indicate that MTX suppresses M1 polarization of RA macrophages, potentially through downregulation of ID2 expression.

### ID2 depletion attenuates LPS-induced M1 polarization of RA macrophages

**Fig. 4:**
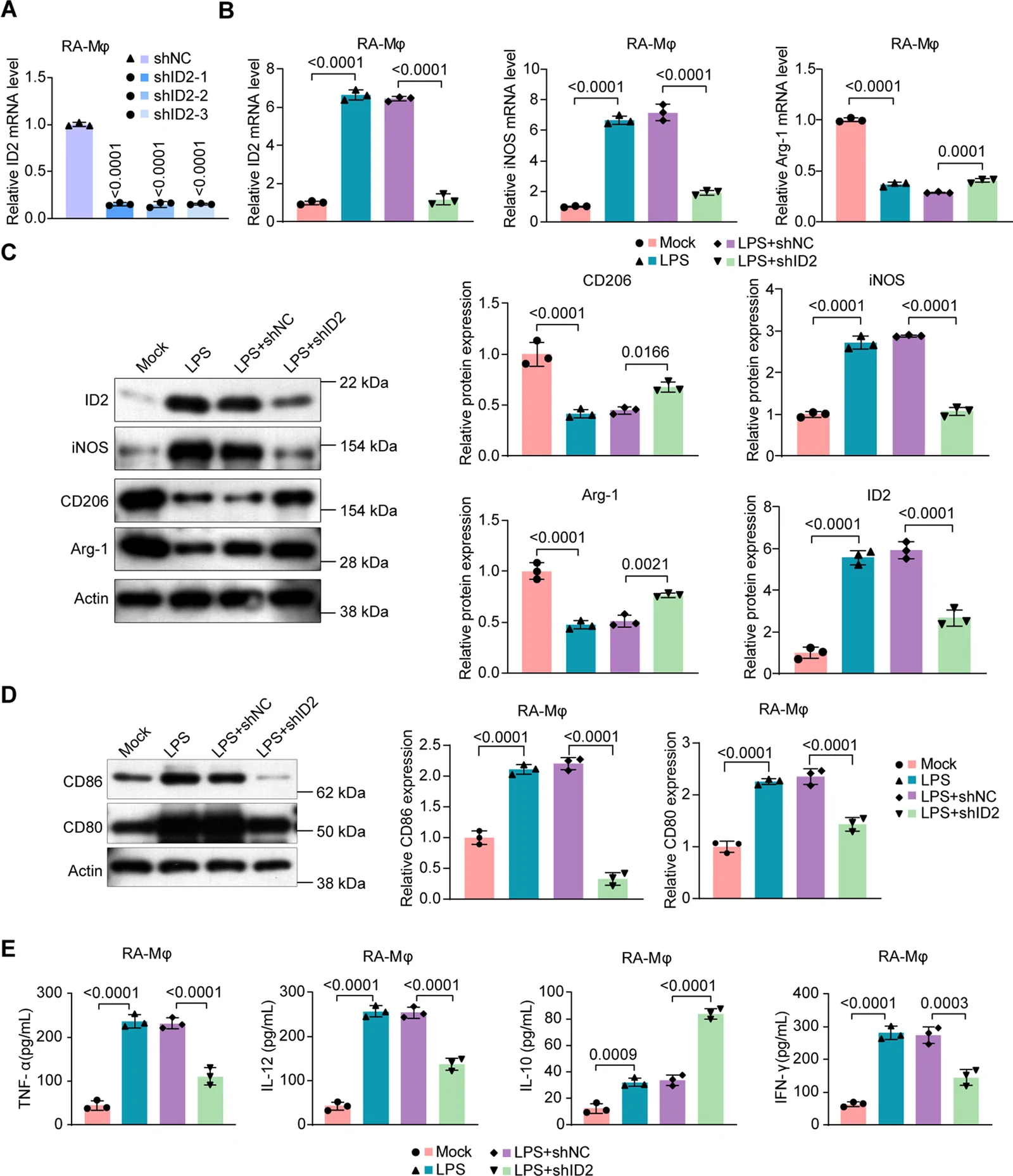
ID2 depletion attenuates M1 polarization of RA macrophages. (**A**) qPCR validation of ID2 knockdown efficiency using three shRNA constructs, and shID2-1 was selected for subsequent experiments. (**B-D**) qPCR and Western blot analyses of ID2 and M1 and M2 polarization markers in LPS-stimulated RA macrophages following ID2 knockdown. (**E**) ELISA quantification of pro- and anti-inflammatory cytokines

To directly assess the role of ID2 in macrophage polarization, ID2 was silenced in RA-Mφs followed by LPS stimulation. qPCR confirmed effective knockdown by all three shID2 constructs, and shID2-1 was selected for subsequent experiments (Fig. 4A). qPCR, Western blot, and ELISA analyses demonstrated that ID2 depletion significantly attenuated LPS-induced M1 polarization, as evidenced by reduced expression of M1 markers iNOS, CD80, and CD86, decreased production of pro-inflammatory cytokines, and enhanced expression of M2 markers Arg-1 and CD206, along with increased IL-10 secretion (Fig. 4B–E). These findings identify ID2 as a positive regulator of M1 polarization in RA macrophages.

### ID2 overexpression counteracts the inhibitory effect of MTX on M1 polarization

**Fig. 5:**
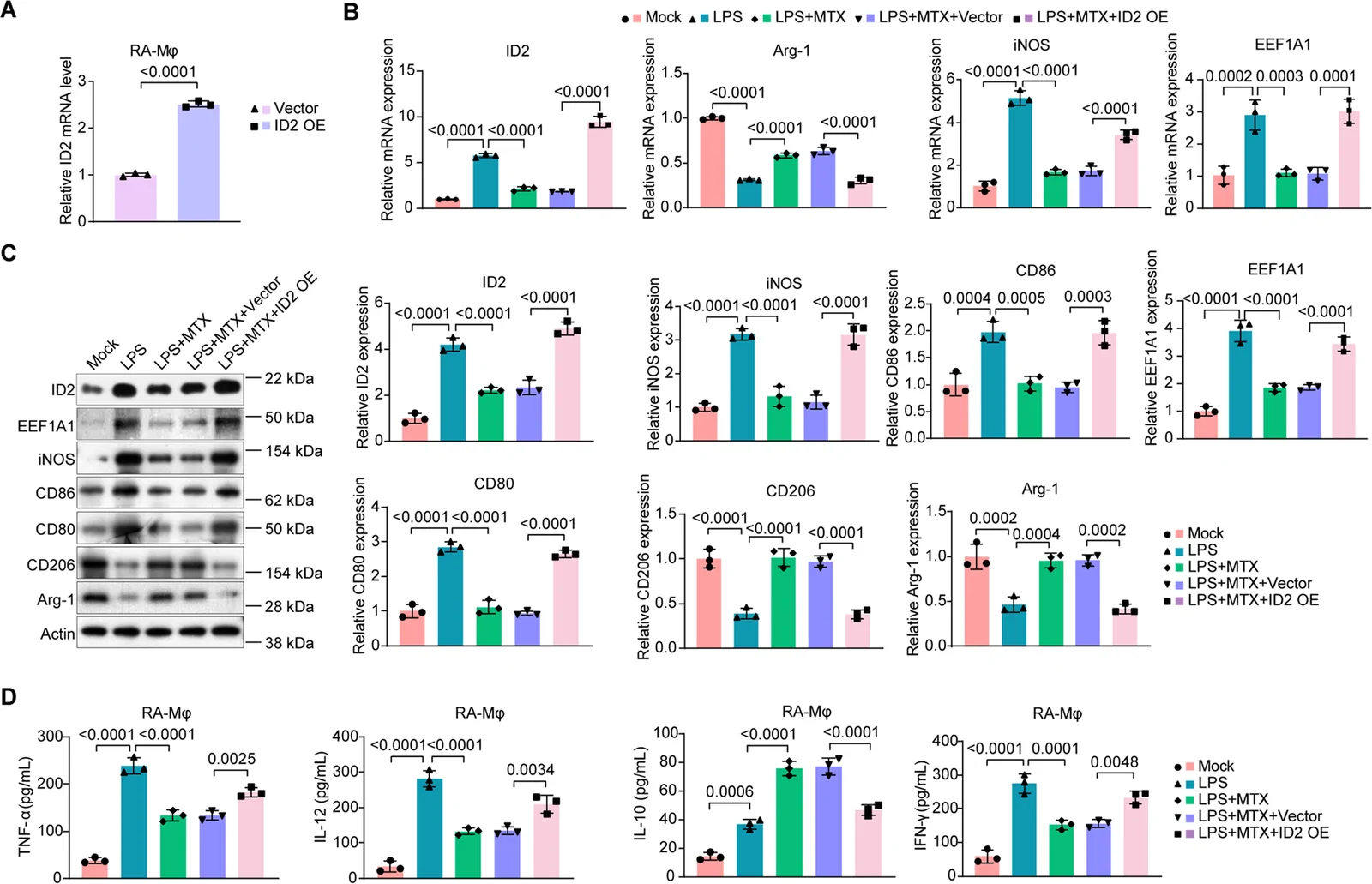
ID2 overexpression counteracts MTX-mediated inhibition of M1 polarization. (**A**) qPCR validation of ID2 overexpression in RA macrophages. (**B**) Expression of iNOS, Arg-1 and EEF1A1 in RA macrophages following ID2 overexpression. (**C**,** D**) qPCR, Western blot, and ELISA analyses showing that ID2 overexpression restores M1 polarization, pro-inflammatory cytokine production expression in LPS-stimulated RA macrophages treated with MTX, while M2 marker expression and IL-10 production are reduced

To investigate whether MTX-mediated suppression of M1 polarization depends on ID2 inhibition, ID2 was overexpressed in RA-Mφs, as confirmed by qPCR (Fig. 5A). Expression analysis of putative ID2 target genes revealed that ID2 overexpression significantly increased iNOS and EEF1A1 levels, whereas Arg-1 expression decreased (Fig. 5B, C).

**Fig. 6:**
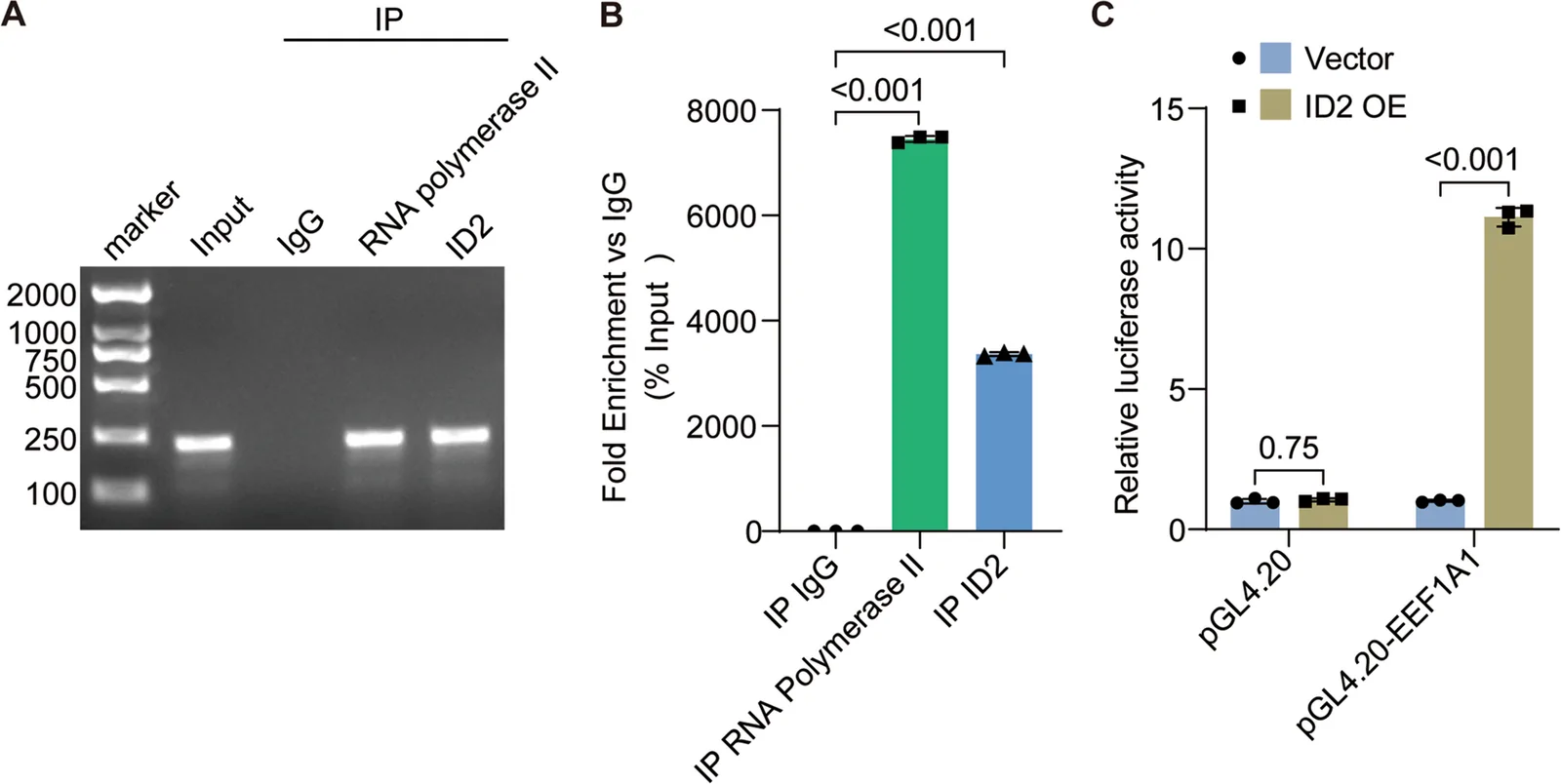
ID2 directly regulates EEF1A1 transcription in RA macrophages. (**A**,** B**) ChIP-RT-PCR and ChIP-PCR showing ID2 binding at the EEF1A1 promoter in RA macrophages; RNA polymerase II serves as a positive control. (**C**) Luciferase reporter assay demonstrating activation of the EEF1A1 promoter by ID2

Subsequently, LPS-stimulated ID2-overexpressing RA-Mφs were treated with MTX. Compared with control cells, ID2-overexpressing macrophages retained higher expression of M1 markers (iNOS, CD80, CD86) and pro-inflammatory cytokines, along with reduced expression of M2 markers (Arg-1, CD206) and IL-10, despite MTX exposure (Fig. 6C, D). These results indicate that MTX inhibits M1 polarization of RA macrophages at least partially through suppression of ID2.

### ID2 directly regulates the transcription of EEF1A1

To elucidate the molecular mechanisms underlying ID2-mediated regulation of macrophage polarization, ChIP assays were performed. ChIP-PCR and ChIP-RT-PCR demonstrated direct binding of ID2 to the promoter region of EEF1A1 in RA macrophages, comparable to RNA polymerase II binding used as a positive control (Fig. 6A, B). This interaction was further validated by luciferase reporter assays, confirming ID2-dependent activation of the EEF1A1 promoter (Fig. 6C).

**Fig. 7:**
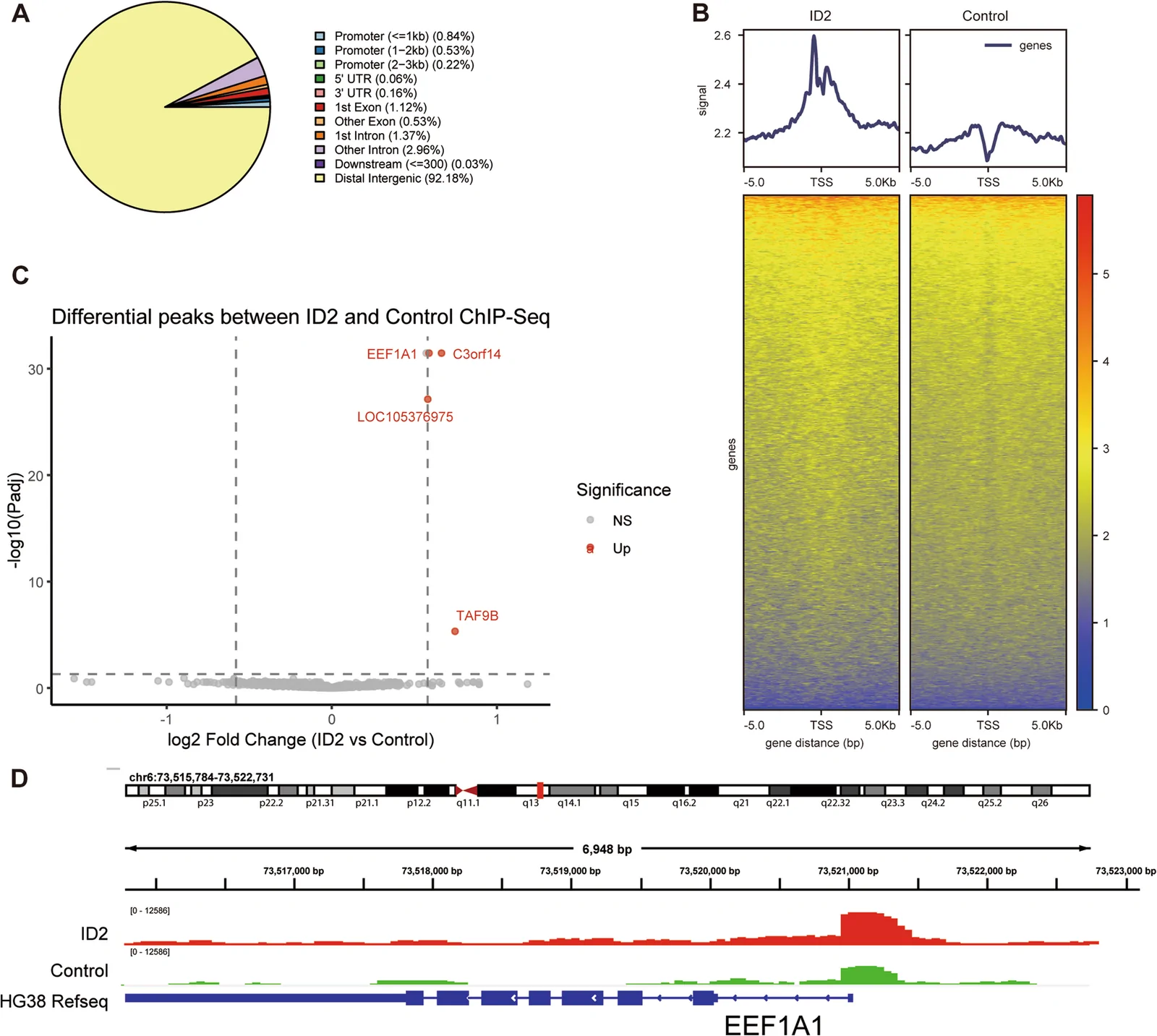
Genome-wide identification of ID2 target genes in RA macrophages by ChIP-seq. (**A**) Distribution of ID2-binding peaks across genomic regions. (**B**) Enrichment of ID2 binding around TSS in control versus ID2-overexpressing RA macrophages. (**C**) Representative ChIP-seq tracks of candidate target genes (EEF1A1, C3orf14, TAF9B, LOC105376975) showing enhanced binding in ID2-overexpressing cells. (**D**) Zoomed view of EEF1A1 promoter showing strong ID2 binding peaks

To comprehensively identify ID2 target genes, ChIP-seq analysis was conducted in ID2-overexpressing RA macrophages. Most ID2-binding peaks were located in distal intergenic regions, followed by intronic, exonic, and promoter regions (Fig. 7A). ID2 overexpression resulted in enhanced signal intensity around transcription start sites (TSSs) compared with control cells (Fig. 7B). Several candidate target genes, including EEF1A1, C3orf14, TAF9B, and LOC105376975, displayed increased binding peaks in ID2-overexpressing cells, with particularly strong enrichment at the EEF1A1 promoter (Fig. 7C, D). These data demonstrate that ID2 directly regulates the transcription of EEF1A1, thereby influencing macrophage polarization and MTX responsiveness in RA.

## Discussion

MTX remains the first-line DMARD for RA, yet a substantial proportion of patients exhibit incomplete or absent clinical responses. The mechanisms underlying MTX resistance remain incompletely understood, particularly with respect to innate immune regulation. In this study, we integrated single-cell transcriptomics with functional and mechanistic analyses to show that altered macrophage polarization, associated with the transcriptional regulator ID2, may contribute to MTX responsiveness in RA.

Our scRNA-seq analysis revealed that while the overall composition of circulating immune cells was largely comparable between MTX-responsive and MTX-unresponsive patients, macrophage-like monocyte from MTX-unresponsive individuals displayed a pronounced pro-inflammatory M1 polarization signature. This observation aligns with previous reports highlighting macrophages as central drivers of synovial inflammation and joint destruction in RA [12, 13]. Importantly, our data extend these findings by demonstrating that monocyte/macrophage polarization status is linked not only to disease activity but also to therapeutic response, suggesting that macrophage-intrinsic programs contribute to MTX resistance.

In our scRNA-seq analysis, the relatively small size of the naïve CD4^+^ T-cell cluster and its separation from the CD8⁺ T-cell cluster in the UMAP analysis are in line with observations from previous studies of PBMCs from RA patients [29, 30]. However, another study reported differing results. Binvignat et al. observed no decrease in naïve CD4⁺ T cells, but instead an expansion of CD4⁺ effector memory T cells, suggesting a shift in T-cell composition [31]. Taken together, these findings indicate that the proportion of naïve CD4⁺ T cells in RA PBMCs may vary across cohorts and experimental platforms, but is generally consistent with a redistribution toward more differentiated T-cell states. The observed UMAP separation may reflect the underlying transcriptomic heterogeneity and systemic immune activation status of T-cell subsets in RA. Besides, a small neutrophil-like population was detected in the PBMC dataset, which may reflect the presence of low-density neutrophils reported in inflammatory conditions such as RA [32], as well as minor technical carryover during PBMC isolation.

Further subclustering identified distinct macrophage-like monocyte subpopulations with differential abundance between MTX-responsive and MTX-unresponsive patients. In particular, PLBD1⁺ and CDKN1A⁺ subcluster were enriched in MTX-unresponsive patients, whereas RLIM⁺ macrophage-like monocyte was relatively depleted. Pseudotime trajectory analysis suggested that these subsets occupy distinct differentiation states, implying that MTX responsiveness may be influenced by both macrophage maturation status and inflammatory programming. These findings underscore the heterogeneity of macrophage-like monocyte in RA and suggest that selective expansion of pro-inflammatory macrophage states may undermine MTX efficacy.

Notably, PLBD1 expression has been reported in airway alveolar macrophages [33], and its expression in glioma-associated macrophages within the tumor microenvironment is associated with patient outcomes [34]. CDKN1A, also known as p21, is a well-established marker of cellular senescence, and CDKN1A⁺ macrophages have been observed within senescence-associated inflammatory niches [35]. RLIM is an E3 ubiquitin ligase that regulates transcriptional multiprotein complexes across diverse biological processes [36]. However, the precise roles of these genes in macrophage function and polarization, as well as the biological relevance of these bioinformatically defined macrophage subpopulations, remain to be determined.

Through integrative trajectory and differential expression analyses, we identified ID2 as a candidate regulator associated with MTX resistance. ID2 expression was broadly reduced in macrophage-like monocyte from MTX-responsive patients and showed strong associations with M1-biased transcriptional programs. While ID2 has been previously studied in T cells, its role in macrophage polarization in RA has remained largely unexplored. Our findings position ID2 as a central node linking macrophage inflammatory state to MTX responsiveness.

Functional experiments using RA-derived macrophages further supported this conclusion. We found that MTX suppressed LPS-induced M1 polarization and pro-inflammatory cytokine production, concomitant with reduced ID2 expression. Conversely, ID2 depletion dampened M1 polarization, whereas ID2 overexpression antagonized the inhibitory effects of MTX on macrophage activation. These results suggest that ID2 acts as a positive regulator of M1 polarization in RA macrophages and that MTX exerts its anti-inflammatory effects, at least in part, by repressing ID2 expression. It should be noted that PBMCs from RA patients may be influenced by the systemic inflammatory milieu and exhibit a primed phenotype. As in vitro experiments were performed using cells from RA patients under comparable experimental conditions, the relative differences observed are less likely to be confounded by baseline inflammatory priming. Nevertheless, this represents a limitation of the study, and inclusion of healthy controls in future studies would further strengthen the conclusions.

Mechanistically, we demonstrated that ID2 directly binds to and transcriptionally regulates EEF1A1. EEF1A1, beyond its canonical role in protein translation, has been increasingly recognized as a modulator of cytoskeletal dynamics and inflammatory responses in immune cells [37–40]. Our ChIP-qPCR, luciferase reporter, and ChIP-seq analyses collectively establish these genes as direct ID2 targets, providing a mechanistic link between ID2 activity and macrophage functional states. Nevertheless, the precise function of these genes in macrophages warrants further investigation.

ID2 expression is unlikely to be restricted to LPS stimulation. Multiple studies have reported associations between ID2 and inflammatory signaling in different cellular contexts. For example, exogenous TNF-α has been shown to upregulate ID2 expression in oral squamous cell carcinoma cell lines, where it contributes to inflammation-associated tumor progression [41]. In addition, Ren et al. [42] demonstrated that ID2 modulates inflammatory responses in LPS-stimulated neutrophils by regulating pro-inflammatory cytokine production and NF-κB signaling. In RA, IFN-γ expression has been reported to correlate with ID2 levels, and inhibition of ID2 suppresses IFN-γ production and disease progression [19]. While these findings suggest that ID2 is involved in inflammatory processes, they do not directly demonstrate cytokine-mediated regulation of ID2 in macrophages. Therefore, the effects of specific cytokines, such as IFN-γ and IL-6, on ID2 expression in macrophages remain to be further investigated. Future studies under diverse cytokine stimulation conditions will be important to clarify the regulatory network of ID2 in RA.

This study has several limitations. The sample size for scRNA-seq was relatively small, and there was a lack of healthy and disease control groups, which limits our ability to determine whether the observed phenotypes are disease-specific. Additionally, PBMC-derived macrophages from RA patients may be influenced by the systemic inflammatory milieu and exhibit a primed phenotype, which could affect their differentiation and functional responses. Future studies incorporating appropriate control cohorts will be necessary to address this issue. In addition, while our in vitro experiments support a role for ID2 in macrophage polarization, in vivo validation using RA animal models will be important to confirm its contribution to disease progression and therapeutic response. Furthermore, the upstream signals regulating ID2 expression in RA macrophages remain to be elucidated. Finally, other potential ID2-regulated genes and their functional roles in macrophage polarization and RA pathogenesis were not explored.

## Conclusions

In summary, our study identifies ID2 as a regulator of macrophage polarization and MTX responsiveness in RA. By integrating single-cell profiling with functional and mechanistic analyses, we reveal an ID2-associated transcriptional network that may contribute to pro-inflammatory macrophage states and reduced MTX efficacy. These findings provide new insights into the mechanisms of MTX resistance and may inform the development of precision-guided therapeutic strategies in RA.

## Supplementary Information

Below is the link to the electronic supplementary material.

Supplementary file1 (DOCX 19.2 KB)

Supplementary figure 1 Comparison of PBMC composition between MTX-responsive and MTX-unresponsive RA patients. UMAP plot showing similar distribution patterns of major immune cell populations between R_MTX and UR_MTX patients. (PNG 84.3 KB)

High Resolution Image (TIF 313 KB)

Supplementary figure 2 Characterization of macrophage subpopulations and pseudotime trajectories. (A) Dot plot showing signature gene expression across 11 macrophage-like monocytes subpopulations. (B, C) Pseudotime trajectories showing differentiation states of macrophage-like monocytes subpopulations. (PNG 285 KB)

High Resolution Image (TIF 1.23 MB)

Supplementary figure 3 Characterization of macrophage subpopulations and pseudotime trajectories. (A) Dot plot showing signature gene expression across 11 macrophage-like monocytes subpopulations. (B, C) Pseudotime trajectories showing differentiation states of macrophage-like monocytes subpopulations. (PNG 41.5 KB)

High Resolution Image (TIF 256 KB)

## Data Availability

The authors confirm that the data supporting the findings of this study are available within the article and can be obtained from the corresponding authors upon reasonable request.

## References

[1] Jutley, G. S., Z. P. Latif, and K. Raza. 2017. Symptoms in individuals at risk of rheumatoid arthritis. *Best Practice & Research Clinical Rheumatology* 31(1):59–70. 10.1016/j.berh.2017.09.016 29221599

[2] Gravallese, E. M., and G. S. Firestein. 2023. Rheumatoid Arthritis — Common Origins, Divergent Mechanisms. *New England Journal of Medicine* 388(6):529–542. 10.1056/NEJMra2103726 36780677

[3] Mohammed, R. H., and B. S. Bhutta. 2025. *Hand and Wrist rheumatoid arthritis*. Treasure Island (FL): StatPearls. 32809725

[4] Maisha, J. A., H. S. El-Gabalawy, and L. J. O’Neil. 2023. Modifiable risk factors linked to the development of rheumatoid arthritis: evidence, immunological mechanisms and prevention. *Frontiers In Immunology* 14:1221125. Epub 20230912. 10.3389/fimmu.2023.1221125 PMC10520718 37767100

[5] Pradeepkiran, J. A. 2019. Insights of rheumatoid arthritis risk factors and associations. *Journal of Translational Autoimmunity* 2:100012. 10.1016/j.jtauto.2019.100012 PMC7388374 32743500

[6] Almutairi, K. B., J. C. Nossent, D. B. Preen, H. I. Keen, and C. A. Inderjeeth. 2021. The Prevalence of Rheumatoid Arthritis: A Systematic Review of Population-based Studies. *The Journal of Rheumatology* 48(5):669–676. 10.3899/jrheum.200367 33060323

[7] Cubberley, C., and A. Maharaj. 2025. Global RA treatment recommendations: An update from the various international societies. *Best Practice & Research Clinical Rheumatology* 39(1):102019. 10.1016/j.berh.2024.102019 39496528

[8] Radu, A. F., and S. G. Bungau. 2021. Management of Rheumatoid Arthritis: An Overview. *Cells* 10(11). Epub 20211023. 10.3390/cells10112857 PMC8616326 34831081

[9] Yu, J., and P. Zhou. 2020. The advances of methotrexate resistance in rheumatoid arthritis. *Inflammopharmacology* 28(5):1183–1193. Epub 20200805. 10.1007/s10787-020-00741-3 32757110

[10] Lendeckel, U., S. Venz, and C. Wolke. 2022. Macrophages: shapes and functions. *ChemTexts* 8(2):12. Epub 20220310. 10.1007/s40828-022-00163-4 PMC8907910 35287314

[11] Guan, F., R. Wang, Z. Yi, P. Luo, W. Liu, and Y. Xie et al. 2025. Tissue macrophages: origin, heterogenity, biological functions, diseases and therapeutic targets. *Signal Transduction and Targeted Therapy* 10(1):93. 10.1038/s41392-025-02124-y PMC11889221 40055311

[12] Zheng, Y., K. Wei, P. Jiang, J. Zhao, Y. Shan, and Y. Shi et al. 2024. Macrophage polarization in rheumatoid arthritis: signaling pathways, metabolic reprogramming, and crosstalk with synovial fibroblasts. *Frontiers In Immunology* 15:1394108. Epub 20240510. 10.3389/fimmu.2024.1394108 PMC11116671 38799455

[13] Siouti, E., and E. Andreakos. 2019. The many facets of macrophages in rheumatoid arthritis. *Biochemical Pharmacology* 165:152–169. 10.1016/j.bcp.2019.03.029 30910693

[14] Ríos, I., B. López-Navarro, M. Torres-Torresano, B. Soler Palacios, M. Simón-Fuentes, and Á Domínguez-Soto et al. 2023. GSK3β Inhibition Prevents Macrophage Reprogramming by High-Dose Methotrexate. *Journal Of Innate Immunity* 15(1):283–296. Epub 20221114. 10.1159/000526622 PMC10643894 36380627

[15] Municio, C., Á Dominguez-Soto, S. Fuentelsaz-Romero, A. Lamana, N. Montes, and V. D. Cuevas et al. 2018. Methotrexate limits inflammation through an A20-dependent cross-tolerance mechanism. *Annals of the Rheumatic Diseases* 77(5):752–759. 10.1136/annrheumdis-2017-212537 PMC5909749 29431121

[16] Municio, C., B. Soler Palacios, L. Estrada-Capetillo, A. Benguria, A. Dopazo, and E. García-Lorenzo et al. 2016. Methotrexate selectively targets human proinflammatory macrophages through a thymidylate synthase/p53 axis. *Annals of the Rheumatic Diseases* 75(12):2157–2165. 10.1136/annrheumdis-2015-208736 26920997

[17] Wu, H., and Q. Shao. 2019. The role of inhibitor of binding or differentiation 2 in the development and differentiation of immune cells. *Immunobiology* 224(1):142–146. 10.1016/j.imbio.2018.09.006 30340915

[18] Rankin, L., and G. T. Belz. 2011. Diverse roles of inhibitor of differentiation 2 in adaptive immunity. *Clinical And Developmental Immunology* 2011:281569. Epub 20110317. 10.1155/2011/281569 PMC3061294 21437223

[19] Sun, H., J. Miao, K. Zhang, P. Zhang, H. Shen, and J. Wang et al. 2025. Id2 exacerbates the development of rheumatoid arthritis by increasing IFN-γ production in CD4(+) T cells. *Clin Transl Med* 15(3):e70242. 10.1002/ctm2.70242 PMC11885165 40051059

[20] Ishiguro, A., K. S. Spirin, M. Shiohara, A. Tobler, A. F. Gombart, and M. A. Israel et al. 1996. Id2 expression increases with differentiation of human myeloid cells. *Blood* 87(12):5225–5231. 8652837

[21] Sato, Y., A. Jain, S. Ohtsuki, H. Okuyama, I. Sturmlechner, and Y. Takashima et al. 2023. Stem-like CD4(+) T cells in perivascular tertiary lymphoid structures sustain autoimmune vasculitis. *Science Translational Medicine* 15(712):eadh0380. Epub 20230906. 10.1126/scitranslmed.adh0380 PMC11131576 37672564

[22] Zheng, Y., Z. Chen, Y. Han, L. Han, X. Zou, and B. Zhou et al. 2020. Immune suppressive landscape in the human esophageal squamous cell carcinoma microenvironment. *Nature Communications* 11(1):6268. Epub 20201208. 10.1038/s41467-020-20019-0 PMC7722722 33293583

[23] Zhang, J., T. Liu, Y. Duan, Y. Chang, L. Chang, and C. Liu et al. 2023. Single-cell analysis highlights a population of Th17-polarized CD4(+) naïve T cells showing IL6/JAK3/STAT3 activation in pediatric severe aplastic anemia. *Journal Of Autoimmunity* 136:103026. Epub 20230329. 10.1016/j.jaut.2023.103026 37001436

[24] Ren, X., W. Wen, X. Fan, W. Hou, B. Su, and P. Cai et al. 2021. COVID-19 immune features revealed by a large-scale single-cell transcriptome atlas. *Cell *184 (7):1895 – 913.e19. 10.1016/j.cell.2021.01.053. Epub 20210203. PMC7857060 33657410

[25] Salcher, S., G. Sturm, L. Horvath, G. Untergasser, C. Kuempers, and G. Fotakis et al. 2022. High-resolution single-cell atlas reveals diversity and plasticity of tissue-resident neutrophils in non-small cell lung cancer. *Cancer Cell* 40(12):1503–20e8. Epub 20221110. 10.1016/j.ccell.2022.10.008 PMC9767679 36368318

[26] Cheng, S., Z. Li, R. Gao, B. Xing, Y. Gao, and Y. Yang et al. 2021. A pan-cancer single-cell transcriptional atlas of tumor infiltrating myeloid cells. *Cell* 184(3):792–809e23. 10.1016/j.cell.2021.01.010 33545035

[27] Xie, B., L. W. Tian, C. Liu, J. Li, X. Tian, and R. Zhang et al. 2025. Disruption of the eEF1A1/ARID3A/PKC-δ Complex by Neferine Inhibits Macrophage Glycolytic Reprogramming in Atherosclerosis. *Adv Sci (Weinh)* 12(15):e2416158. Epub 20250220. 10.1002/advs.202416158 PMC12005739 39973763

[28] Choudhury, A., D. Bullock, A. Lim, J. Argemi, P. Orning, and E. Lien et al. 2020. Inhibition of HSP90 and Activation of HSF1 Diminish Macrophage NLRP3 Inflammasome Activity in Alcohol-Associated Liver Injury. *Alcoholism, Clinical And Experimental Research* 44(6):1300–1311. Epub 20200518. 10.1111/acer.14338 PMC7328660 32282939

[29] Wu, X., Y. Liu, S. Jin, M. Wang, Y. Jiao, and B. Yang et al. 2021. Single-cell sequencing of immune cells from anticitrullinated peptide antibody positive and negative rheumatoid arthritis. *Nature Communications* 12(1):4977. 10.1038/s41467-021-25246-7 PMC8371160 34404786

[30] Gui, L., X. Luo, L. Zhou, Q. Wei, and J. Gu. 2023. Peripheral CD4+ /CD8 + T cell composition distinct from healthy individuals is shared by ankylosing spondylitis and rheumatoid arthritis. *International Journal Of Rheumatic Diseases* 26(10):2014–2023. 10.1111/1756-185X.14860 37635355

[31] Binvignat, M., B. Y. Miao, C. Wibrand, M. M. Yang, D. Rychkov, and E. Flynn et al. 2024. Single-cell RNA-Seq analysis reveals cell subsets and gene signatures associated with rheumatoid arthritis disease activity. *JCI Insight* 9(16):e178499. 10.1172/jci.insight.178499 PMC11343607 38954480

[32] Bhargav, A., V. Kumar, and N. K. Rai. 2025. Roles of Neutrophils in Autoimmune Diseases and Cancers. *International Journal Of Molecular Sciences* 26(18):9040. Epub 20250917. 10.3390/ijms26189040 PMC12469507 41009604

[33] Gibbings, S. L., R. Goyal, A. N. Desch, S. M. Leach, M. Prabagar, and S. M. Atif et al. 2015. Transcriptome analysis highlights the conserved difference between embryonic and postnatal-derived alveolar macrophages. *Blood* 126(11):1357–1366. Epub 20150731. 10.1182/blood-2015-01-624809 PMC4566811 26232173

[34] Wei, M., G. Zhou, L. Chen, Y. Zhang, W. Ma, and L. Gao et al. 2024. The prognostic and immune significance of PLBD1 in pan-cancer and its roles in proliferation and invasion of glioma. *Journal Of Cancer* 15(12):3857–3872. Epub 20240520. 10.7150/jca.96365 PMC11190780 38911364

[35] Young, L. V., W. Morrison, C. Campbell, E. C. Moore, M. G. Arsenault, and A. G. Dial et al. 2021. Loss of dystrophin expression in skeletal muscle is associated with senescence of macrophages and endothelial cells. *American Journal of Physiology-Cell Physiology* 321(1):C94–C103. 10.1152/ajpcell.00397.2020 33979211

[36] Jiao, B., N. Taniguchi-Ishigaki, C. Güngör, M. A. Peters, Y. W. Chen, and S. Riethdorf et al. 2013. Functional activity of RLIM/Rnf12 is regulated by phosphorylation-dependent nucleocytoplasmic shuttling. *Molecular Biology Of The Cell* 24(19):3085–3096. Epub 20130731. 10.1091/mbc.E13-05-0239 PMC3784382 23904271

[37] Vera, M., B. Pani, L. A. Griffiths, C. Muchardt, C. M. Abbott, and R. H. Singer et al. 2014. The translation elongation factor eEF1A1 couples transcription to translation during heat shock response. *eLife* 3:e03164. 10.7554/eLife.03164 PMC4164936 25233275

[38] Katow, H., and H. D. Ryoo. 2024. eEF1α2 is required for actin cytoskeleton homeostasis in the aging muscle. *Disease Models & Mechanisms* 17 (9). Epub 20240829. 10.1242/dmm.050729 PMC11381931 39207054

[39] Xing, L., T. Wu, H. Xu, S. Yang, C. Luan, and Y. Xu et al. 2025. Single-Cell Transcriptomics Uncover EEF1A1-Driven Ubiquitination Dysregulation in T Cell Exhaustion and SLE Pathogenesis via STAT1-Mediated Th1/Th2 Imbalance. *Mediators Inflamm* 2025:3708640. Epub 20251111. 10.1155/mi/3708640 PMC12626703 41262540

[40] Schulz, I., C. Engel, A. J. Niestroj, A. Kehlen, J-U. Rahfeld, and M. Kleinschmidt et al. 2014. A non-canonical function of eukaryotic elongation factor 1A1: Regulation of interleukin-6 expression. *Biochimica et Biophysica Acta (BBA) - Molecular Cell Research* 1843(5):965–975. 10.1016/j.bbamcr.2014.01.022 24487064

[41] Zhou, J. P., Z. L. Gao, M. L. Zhou, M. Y. He, X. H. Xu, and D. T. Tao et al. 2015. Snail interacts with Id2 in the regulation of TNF-α-induced cancer cell invasion and migration in OSCC. *Am J Cancer Res* 5(5):1680–1691. PMC4497435 26175937

[42] Ren, J., D. Yan, Y. Wang, J. Zhang, M. Li, and W. Xiong et al. 2021. Inhibitor of Differentiation-2 Protein Ameliorates DSS-Induced Ulcerative Colitis by Inhibiting NF-κB Activation in Neutrophils. *Frontiers In Immunology* 12:760999. Epub 2021/11/23. 10.3389/fimmu.2021.760999 PMC8599958 34804049

