# Supplementary material for: ID2 Regulates Macrophage Polarization and Mediates Methotrexate Responsiveness in Rheumatoid Arthritis
Source: Inflammation. 2026 May 22;49(1):217. doi: 10.1007/s10753-026-02527-1 (PMC13755079; doi:10.1007/s10753-026-02527-1)
Supplement: Supplementary file 1 [file 10753_2026_2527_MOESM1_ESM.docx]

**Supplementary Table S1. Clinical characteristics of RA patients used for scRNA-seq analysis.**

| Characteristics | MTX-responsive patients | | | MTX-unresponsive patients | | |
| --- | --- | --- | --- | --- | --- | --- |
| Patient ID | Patient 1 | Patient 2 | Patient 3 | Patient 4 | Patient 5 | Patient 6 |
| Age (years) | 55 | 60 | 62 | 50 | 61 | 63 |
| Gender | Female | Female | Female | Female | Female | Male |
| Disease duration (months) | 11 | 9 | 6 | 8 | 5 | 7 |
| **Treatment** |  |  |  |  |  |  |
| MTX (mg) | 15 | 15 | 15 | 15 | 15 | 20 |
| NSAIDs | Celecoxib | Meloxicam | Loxoprofen | Etoricoxib | Meloxicam | Loxoprofen |
| **DAS28-CRP** |  |  |  |  |  |  |
| Pre-treatment | 3.63 | 3.80 | 3.98 | 3.90 | 3.48 | 4.13 |
| Post-treatment | 2.22 | 2.36 | 2.71 | 3.80 | 3.32 | 3.63 |

DAS28-CRP, disease activity score of 28 joints based on C-reactive protein; MTX, Methotrexate; NSAIDs, nonsteroidal anti-inflammatory drugs; RA, rheumatoid arthritis.

**Supplementary** **Table S2.** **Clinical characteristics of RA patients used for macrophage differentiation experiments.**

| Characteristics | Patient 1 | Patient 2 | Patient 3 |
| --- | --- | --- | --- |
| Age (years) | 57 | 59 | 65 |
| Gender | Female | Female | Female |
| Disease duration (months) | 5 | 3 | 6 |
| **Treatment** |  |  |  |
| NSAIDs | Imrecoxib | Meloxicam | Diclofenac |
| **DAS28-CRP** | 3.58 | 3.72 | 3.97 |

DAS28-CRP, disease activity score of 28 joints based on C-reactive protein; NSAIDs, nonsteroidal anti-inflammatory drugs; RA, rheumatoid arthritis.
